# A putative *Mycobacterium tuberculosis* glyoxalase Rv0801 promotes bacterial fitness by alleviating methylglyoxal stress and blunts NRF2-mediated antioxidant defenses

**DOI:** 10.3389/fimmu.2026.1745502

**Published:** 2026-02-27

**Authors:** Haiqi Chen, Qi’ao Zhang, Wei Wu, Xinyi He, Abulimiti Abudukadier, Yun Qi, Qun Sun, Peibo Li, Jianping Xie

**Affiliations:** 1 Institute of Modern Biopharmaceuticals, School of Life Sciences, Southwest University, Chongqing, China; 2 Key Laboratory of Bio‐resources and Eco‐environment of the Ministry of Education, College of Life Sciences, Sichuan University, Chengdu, China; 3 Department of Gynecology & Pediatrics Tuberculosis, Xi'an Chest Hospital, Xi'an, Shaanxi, China; 4 Department of the Tuberculosis, Chongqing Public Health Medical Center, Chongqing, China

**Keywords:** glyoxalase system, KEAP1-NRF2 pathway, macrophage immunity, methylglyoxal (MG), *Mycobacterium tuberculosis*, mycothiol (MSH)

## Abstract

**Introduction:**

Methylglyoxal (MG), a toxic metabolic byproduct, functions as a potent antibacterial weapon deployed by macrophages. The glyoxalase system represents the primary microbial defense against MG, yet its role in *Mycobacterium tuberculosis* pathogenesis remains incompletely defined.

**Methods:**

To define the function of the putative *M. tuberculosis glyoxalase* Rv0801 and its homolog MSMEG_5827, we used genetic engineering in *Mycobacterium smegmatis* MC_2_-155, coupled with growth and macrophage infection assays. Host mechanisms were dissected via transcriptomic and biochemical analysis of the KEAP1-NRF2 antioxidant pathway and pro-inflammatory responses.

**Results:**

We demonstrate that Rv0801, conferring robust MG tolerance in a mycothiol (MSH)-dependent manner, is essential for bacterial fitness under MG stress. Mechanistically, Rv0801 orchestrates a dual-pathway interference within infected macrophages: by detoxifying MG, it suppresses the host KEAP1-NRF2 antioxidant pathway and concurrently dampens immunoprotective responses. This coordinated suppression compromises macrophage-mediated bacterial clearance.

**Discussion:**

These findings establish Rv0801-mediated MG stress management as a critical virulence mechanism and highlight the bacterial glyoxalase as a promising target for tuberculosis therapy.

## Highlights

- A putative *Mycobacterium tuberculosis* glyoxalase Rv0801 confers resistance to methylglyoxal.
- The MG detoxification process mediated by Rv0801 is mycothiol (MSH)-dependent.
- Rv0801 alleviates MG-induced carbonyl and oxidative stress.
- Enhanced MG detoxification of Rv0801 recombinant *M. smegmatis* promotes bacterial fitness in THP-1 macrophages.
- Rv0801 impedes the KEAP1-NRF2 signaling pathway and impairs immunoprotective responses.

## 1 Introduction

Methylglyoxal (MG), a highly reactive byproduct of glycolysis, indiscriminately attacks critical biomolecules, inducing irreversible glycation modifications that trigger structural damage and functional disruption (1). Notably, MG promotes DNA-DNA and DNA-protein crosslinking and covalently binds to lysine or arginine residues of proteins, forming irreversible advanced glycation end-products (AGEs) (2). Accumulation of AGEs has been directly linked to chronic diseases, such as diabetes, neurodegenerative disorders and cancer (3–5).

Host-derived MG and related aldehydes can restrict the survival of intracellular pathogens (6, 7). *Mycobacterium tuberculosis* (Mtb), the causative agent of tuberculosis (TB), must overcome this host-derived carbonyl stress to establish infection.

As the primary host niche for Mtb, the macrophage, undergoes metabolic reprogramming to aerobic glycolysis during infection (8, 9). This transition facilitates immune activation and concurrently elevates intracellular levels of MG (7). This MG-induced stress can activate the host’s central antioxidant regulator, the Kelch-like ECH-associated protein 1-NF-E2-related factor 2 (KEAP1-NRF2) pathway (10, 11). Under homeostasis, the redox sensor KEAP1 acts as an adaptor for a Cullin3-based E3 ubiquitin ligase complex that binds to the Neh2 domain of NRF2, leading to its continuous ubiquitination and proteasomal degradation (12). Upon electrophilic or oxidative challenge such as MG exposure, modification of proximal cysteine and arginine residues on KEAP1 disrupts its repressive function (13). This enables NRF2 stabilization and nuclear translocation, activating a battery of antioxidant and detoxifying genes transcription, including those involved in glutathione (GSH) metabolism (14–16). This response is essential for restoring cellular redox homeostasis and sustaining antimicrobial capacity (17, 18). Thus, Mtb face a dual challenge: it must detoxify MG to survive its direct toxicity while also adapting to the enhanced host antioxidant defense orchestrated by NRF2 activation. How Mtb navigates this integrated stress landscape, and whether its MG detoxification machinery is directly linked to modulating the host’s KEAP1-NRF2 pathway, remains a key unresolved question.

Bacteria employ the conserved glyoxalase system for MG detoxification (19), utilizing GSH to convert toxic MG into harmless D-lactate, which is catalyzed cooperatively by glyoxalase A (GloA, lactoylglutathione lyase) and glyoxalase B (GloB, hydroxyacylglutathione hydrolase) (20), thereby protecting cellular thiol pools and redox homeostasis (21–23). For instance, in *Synechocystis* sp. PCC 6803, *sll1019* and *slr1259* genes have been shown to confer oxidative stress tolerance precisely by enhancing glyoxalase pathway activity and elevating cellular antioxidant levels such as GSH and superoxide dismutase (24). Furthermore, bacterial glyoxalase enzymes can function as virulence factors, modulating host immunity (25).

While *Mycobacterium smegmatis* glyoxalase MSMEG_2975 has been shown to regulate growth and biofilm formation (26), functional insights into Mtb’s glyoxalase are only emerging. The GloA ortholog Rv0577 activates pro-inflammatory pathways via TLR2/MyD88-dependent signaling, thereby directing T-cell polarization toward a Th1 phenotype (27, 28). Concurrently, the glyoxalase Rv0911 demonstrates DNA repair activity under MG stress (7). Notably, *Rv0801* (the Mtb ortholog of *M. smegmatis* MSMEG_5827), encoding a putative glyoxalase, shows the most prominent adaptive signatures under macrophage-induced selective pressures (29). This suggests that Rv0801 plays a critical and specialized role in managing MG-induced stress during infection. To functionally dissect this role, we employed a comparative genetics approach in *M. smegmatis*, a fast−growing non−pathogenic species phylogenetically related to the *Mycobacterium tuberculosis* complex (MTBC) and commonly used as a surrogate in molecular studies. We hypothesized that Rv0801 recombinant strains would exhibit enhanced MG detoxification and improved redox homeostasis under carbonyl stress, whereas strains deficient in its ortholog (MSMEG_5827) would display increased susceptibility. This system allowed us to directly test the contribution of this adaptive gene to bacterial fitness within the integrated stress landscape of MG toxicity and host antioxidant defense.

However, the composition of Mtb glyoxalase system, its regulatory networks, and its multifaceted roles in host-pathogen interactions remain poorly defined. A systematic dissection of its core components and biological functions could not only deepen our understanding of how Mtb leverages metabolic adaptations to counteract host defense mechanisms but also provide critical insights for developing novel anti-tubercular therapies targeting metabolic vulnerabilities.

## 2 Materials and methods

### 2.1 Bacteria and cell culture

*E. coli* DH5α was used for cloning and was cultivated in Luria-Bertani medium. *M. smegmatis* mc^2–^155 was cultured in Middlebrook 7H9 liquid medium supplemented with 0.2% glycerol and 0.05% Tween-80, or in Middlebrook 7H9 solid medium containing 0.2% glycerol. Antibiotics or inducers were used at the following concentrations when required: kanamycin (50 *μ*g/mL for *E. coli*, 25 *μ*g/mL for *M. smegmatis*), hygromycin (150 *μ*g/mL for *E. coli*, 75 *μ*g/mL for *M. smegmatis*), anhydrotetracycline (aTc, 100 ng/mL for *M. smegmatis*) and acetamide (ACE, 28 mM for *M. smegmatis*). Plasmids, strains, and primers are listed in **Supplementary Table S1**. THP-1 cells were cultured in RPMI 1640 medium supplemented with 10% (v/v) heat-inactivated fetal bovine serum (FBS), 2 mM L-glutamine, 100 *μ*g/mL streptomycin, and 100 U/mL penicillin at 37°C with 5% CO_2_.

### 2.2 Construction of strains and plasmids

#### 2.2.1 Recombinant bacterial construction

*Rv0801* was amplified from the genomic DNA of Mtb using primers containing restriction sites and cloned into the pALACE plasmid (induced by ACE) via Gibson assembly to generate pALACE-*Rv0801*. The recombinant plasmid and empty pALACE vector were then electroporated into wild-type *M. smegmatis* (WT_MS) to generate MS_*Rv0801* and MS_pAL strains, respectively.

#### 2.2.2 CRISPRi knockdown strains construction

The CRISPRi plasmid was constructed as previously described (30) using the aTc-inducible backbone from Addgene plasmid #166886. Briefly, the plasmid backbone was linearized with *Bsm*BI-v2 (NEB) and gel-purified. Single-guide RNAs (sgRNAs) targeting the non-template strand within the *MshA* (*MSMEG_0933*) open reading frame (ORF) were designed using the online tool PEBBLE (https://pebble.rockefeller.edu/). For each sgRNA, a pair of complementary oligonucleotides with appropriate sticky-end overhangs was annealed and ligated into the digested plasmid backbone using T4 DNA ligase (Takara). After transformation, clones were selected on kanamycin-containing medium, and correct sgRNA insertion was verified by colony PCR.

#### 2.2.3 Knockout and complemented strains constructions

The *MSMEG_5827* deletion mutant was generated in *M. smegmatis* using a CRISPR-Cas12a (Cpf1)-assisted recombination system (31). First, pJV53-Cpf1 was transformed into *M. smegmatis* to express recombinase and Cpf1 protein. For targeted deletion of *MSMEG_5827*, a crRNA expression plasmid was constructed. Two complementary oligonucleotides containing the *MSMEG_5827*-targeting sequence adjacent to the 5’-YTN-3’ PAM motif were synthesized. The oligonucleotides were annealed to form a protospacer cassette with *Hin*d III and *Bpm* I overhangs at the 3’ and 5’ ends, which was then ligated into the pCR−Hyg plasmid. Simultaneously, homologous arms including 500 bp upstream and downstream of the target gene *MSMEG_5827* were amplified from WT_MS genomic DNA using primers containing regions flanking *MSMEG_5827* by overlap PCR. Homologous fragments and crRNA expression plasmid were co-electroporated into *M. smegmatis* cells harboring pJV53-Cpf1. After recovery, transformants were selected on 7H10 agar plates supplemented with hygromycin (50 *µ*g/mL), kanamycin (25 *µ*g/mL), and aTc (100 ng/mL) (to induce Cpf1 expression). Successful deletion mutants were verified by colony PCR and sequencing. For genetic complementation, a DNA fragment containing 500 bp upstream of *MSMEG_5827* (including its native promoter) was amplified and cloned into pHY plasmid via Gibson assembly to generate pHY-*MSMEG_5827.* The resulting plasmid was then transformed into the Δ*MSMEG_5827* mutant strain to produce the complemented strain Δ*MSMEG_5827*(pHY-*MSMEG_5827*).

### 2.3 Growth curves

Growth curves of wild-type and mutant *M. smegmatis* strains were determined by spectrophotometry (UV-VIS spectrophotometer, Varian Cary 50). Briefly, overnight cultures grown to mid-late exponential phase were harvested by centrifugation at 6,000 rpm for 10 min, washed twice with 1×phosphate-buffered saline (PBS), and adjusted to an optical density at 600 nm (OD_600_) of 0.8 (approximately 1 × 10^8^ cells mL^-1^). A 1% (v/v) inoculum was transferred into fresh 7H9 medium containing either 0.25 mM MG (Macklin) or no additive (untreated control). Cultures were incubated at 37°C with shaking (110 rpm). Where indicated, GSH was added to a final concentration of 0.25 mM at 12 h post-inoculation. The OD_600_ was measured every 4 h for 48 h.

### 2.4 Methylglyoxal susceptibility assay

Cultures were prepared as in section 2.3. Cells were then diluted 100−fold in fresh 7H9 medium (10 mL final volume), and treated with 0, 0.5, 1 or 2 mM MG in triplicate. These concentrations were selected based on pilot experiments that revealed differential tolerance between Rv0801 recombinant strains and Δ*MSMEG_5827* strains. To evaluate protection against MG toxicity, GSH was added to 7H9 agar plates at equimolar concentrations relative to MG, reflecting the 1:1 stoichiometry of the spontaneous MG-GSH reaction. At indicated time points (4, 8, 12 or 24 h post-treatment), 1 mL of culture was collected, washed with 1×PBS and serially diluted (10^-1^ to 10^-5^) onto plates with or without GSH. Viability was determined by counting colony−forming units (CFUs) after 3 days of incubation at 37°C.

### 2.5 H_2_O_2_ susceptibility assay

Bacterial cultures were prepared as in section 2.3 and then exposed to 0.07 mM H_2_O_2_ in 7H9 agar plates, while control cultures were left untreated. This concentration was selected based on a previous study (32) and further optimized in pilot experiments. Cell viability was assessed by performing serial dilutions, followed by spotting 10 *μ*L aliquots (in triplicate) onto 7H9 agar plates. Viability was determined by CFUs after 3 days of incubation at 37°C.

### 2.6 pH stress survival assay

Bacterial cultures were prepared as in section 2.3 and the cells were then resuspended and normalized to an OD_600_ of 0.8 using 7H9 liquid medium that had been pre-adjusted to pH 4.5, 5.5, 6.5, or 7.5, with a final volume of 10 mL per condition. Cultures were incubated at 37°C with shaking (110 rpm). At 3, 6 or 9 h post-treatment, 1 mL aliquots were collected, washed, and resuspended in 1 mL of fresh PBS. Bacterial viability was assessed by preparing 10-fold serial dilutions in PBS and spotting 10 *μ*L aliquots onto 7H9 agar plates. Viability was determined by CFUs after 3 days of incubation at 37°C. All experiments were performed independently at least three times.

### 2.7 ROS detection

Bacteria or cultured cells were grown under standard conditions (e.g., 7H9 liquid medium for bacteria, RPMI 1640 medium for THP-1 cells) until they reached the logarithmic growth phase (OD_600_ of 0.6 for bacteria; 70-80% confluence for adherent cells). Bacterial strains were then treated with 2 mM MG for 3 h at 37°C prior to analysis. THP-1 cells were infected by different strains for 24 h. Intracellular ROS levels were quantified using the Reactive Oxygen Species Assay Kit (Solarbio), following the manufacturer’s protocol based on the fluorescent probe 2’,7’-dichlorofluorescin diacetate (DCFH-DA).

### 2.8 Rifampin antibiotic susceptibility assay

Bacterial cultures were prepared as in section 2.3. Antibiotic sensitivity was assessed by a spot dilution assay in which serial 10−fold dilutions of the cultures were spotted onto 7H9 agar plates containing 10 *μ*g/mL rifampicin. This concentration was selected based on our experimental system (33). Plates were incubated at 37°C for 3–5 days, and viability was determined by CFUs. Each experiment was performed independently at least three times to ensure reproducibility.

### 2.9 Combined stress assay with methylglyoxal and rifampicin

Cultures were prepared as in section 2.3 and then diluted 10−fold in 7H9 liquid medium (10 mL final volume) containing either 1 mM MG or no additive (untreated control), followed by incubation at 37°C with shaking (110 rpm). After 4 h post-treatment, 1 mL aliquots were collected, centrifuged (4000 rpm, 5 min), and the pellet was washed twice with 1×PBS. Cells were then resuspended in 1 mL of fresh 1×PBS and subjected to 10−fold serial dilutions. A 10 *μ*L aliquot from each dilution was spotted onto 7H9 agar plates supplemented with or without 5 *μ*g/mL rifampicin. Plates were incubated at 37°C for 3-4 days, and bacterial viability was assessed by enumerating CFUs.

### 2.10 *In vitro* macrophage infection assay

THP-1 cells were cultured in RPMI 1640 medium supplemented with 10% (v/v) heat-inactivated FBS, 2 mM L-glutamine, 100 *μ*g/mL streptomycin, and 100 U/mL penicillin at 37°C with 5% CO_2_. Cells were seeded at a density of 1 × 10^6^ cells/mL in 12-well plates and differentiated using 100 ng/mL phorbol 12-myristate 13-acetate (PMA). Before infection, differentiated macrophages were pre-treated as follow: (i) with 0.1 mM MG or 25 mM glucose (Macklin) for 4 h; or (ii) with 5 *μ*M CBR−470−1, 5 mM 2-deoxy-D-glucose (2−DG), or 2 mM N−acetylcysteine (NAC) (MedChemExpress) for 18 h. Cells were then infected with MS_pAL or MS_*Rv0801* at a multiplicity of infection (MOI) of 10. At 4 h post-infection, cells were washed with PBS, and incubated with complete medium containing gentamicin (100 *μ*g/mL) to eliminate extracellular bacteria. To evaluate the effect of exogenous GSH, 0.1 mM GSH was added to designated wells immediately after the washing step. For assessment of intracellular bacterial survival, infected THP-1 cells were lysed at 6, 12, 24, 36 and 48 h post-infection. Cells were washed three times with PBS, lysed in 1 mL of 0.025% sodium dodecyl sulfate (SDS), serially diluted, and plated onto 7H9 agar plates. Bacterial viability was determined by counting CFUs after 3 days of incubation at 37°C.

### 2.11 CCK-8 cell viability assay

THP-1 cells were seeded in a 96-well plate at a density of 1×10^4^ cells per well in 100 *µ*L of complete medium and cultured for 48 h. Cells were then infected separately with either MS_*Rv0801* or MS_pAL at a MOI of 10 for 24 hours. Following infection, cell viability was assessed using a Cell Counting Kit-8 (CCK-8). Briefly, after pre-warming the microplate reader for 30 minutes, 10 *μ*L of CCK-8 solution was added to each well and the plate was incubated for an additional 2 h at 37°C. The absorbance at 450 nm was then measured using the microplate reader. Cells viability was calculated relative to uninfected control cells.

### 2.12 Determination of GSH content

THP-1 cells were collected at 24 h post-infection. Intracellular GSH content was determined using a Reduced Glutathione Content Assay Kit (Solarbio), following the manufacturer’s instructions.

### 2.13 Quantitative real-time PCR

To assess the mRNA levels of *MSMEG_5827*, *MshA*, *MshB*, *MshC*, and *MshD* under MG exposure, overnight cultures of *M. smegmatis* were grown to mid-exponential phase. Cells were harvested, washed twice with PBS, and resuspended in 7H9 medium (containing 0.05% Tween 80) to an OD_600_ of 1.0. WT_MS was then treated with 2 mM MG or left untreated for 3 h at 37°C. For infection assays, PMA-differentiated THP-1 cells were pre-treated under the following conditions prior to infection: with 0.1 mM MG for 4 h; with 5 *μ*M CBR−470−1 or 5 mM 2−DG for 18 h; or left untreated as a control. Subsequently, cells were infected with either MS_pAL or MS_*Rv0801* at a MOI of 10 for 24 h. Total RNA from all samples was isolated using an RNA extraction kit (Promega), and cDNA was synthesized with a reverse transcription kit (Takara). All samples were prepared in three biological replicates. Quantitative real-time PCR was performed on a CFX96 Real-Time PCR Detection System (Bio-Rad) using SYBR Green Master Mix (Takara). For bacterial samples, gene expression was normalized to *rpoB* (*MSMEG_1367*). For THP-1 cell samples, gene expression was normalized to β-actin. Relative mRNA expression for all samples was calculated using the 2^−ΔΔCt^ method (34). Primer sequences are provided in **Supplementary Table S1**.

### 2.14 Western blot

THP-1 cells were infected with different bacterial strains at a MOI of 10. After 24 h of infection, cells were washed three times with PBS, and lysed using RIPA lysis buffer to collect total protein. The protein was separated by 12% SDS-PAGE and transferred onto a nitrocellulose (NC) membrane at 15 V for 1-2 h, with the transfer duration adjusted according to protein size. Membranes were blocked with 5% bovine serum albumin (BSA) in TBST (Tris-buffered saline with Tween-20) for 2 hours at room temperature on a shaker. Then membranes were incubated overnight at 4°C with primary antibodies against β-Actin, Nqo1 and Nrf2 (Abmart). After five washes with TBST, membranes were incubated with horseradish peroxidase (HRP)-conjugated goat anti-mouse or goat anti-rabbit secondary antibodies (Beyotime Biotechnology) for 2 hours at room temperature, then were washed five times with TBST. Protein bands were visualized using an ECL chemiluminescence detection kit (Vazyme). Quantitative analysis of the bands was carried out with ImageJ.

### 2.15 Statistics and reproducibility

Data are presented as the mean ± standard error of the mean (SEM) from at least three independent experiments. Statistical comparisons between two groups were performed using the two-tailed Student’s *t*-test. Data visualization and statistical analysis were conducted using GraphPad Prism 9.3. Differences were considered statistically significant at *P < 0.05, **P < 0.01, and ***P < 0.001.

## 3 Results

### 3.1 Rv0801 functions as a potential glyoxalase to detoxify MG

**Figure 1:**
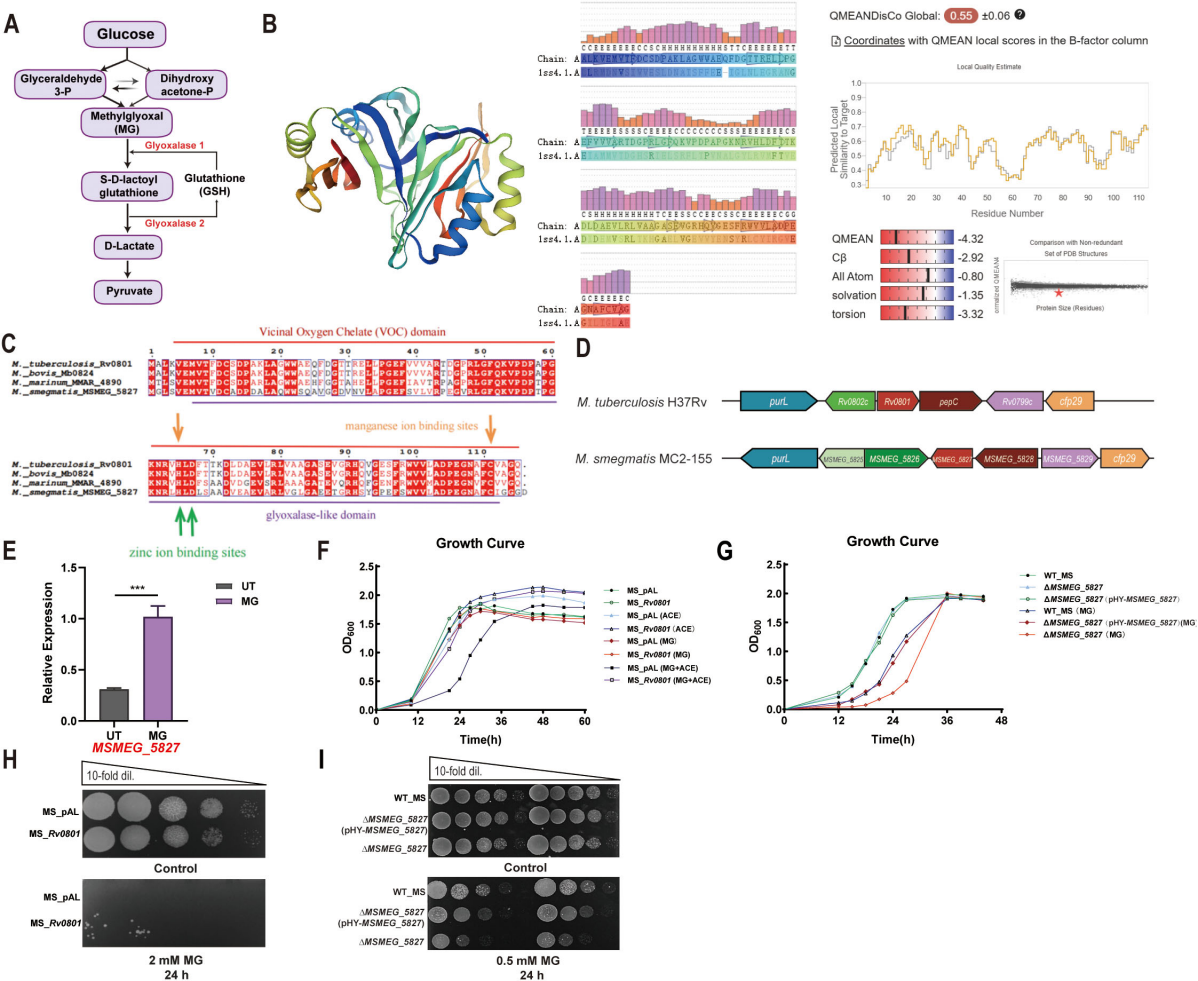
Rv0801 functions as a putative glyoxalase and protects against MG toxicity. **(A)** Schematic of MG formation from glycolytic triose phosphates and its canonical detoxification via the GSH-dependent glyoxalase pathway. **(B)** A homology model of Rv0801 from *Mycobacterium tuberculosis* was generated using the *SWISS-MODEL* server. The crystal structure (1ss4.1.A) of a glyoxalase family protein from *Bacillus cereus* was selected as the template based on its sequence similarity to Rv0801. The homology model provides structural insights into the Rv0801 protein product and indicates its molecular function as a putative glyoxalase. **(C)** Conservation analysis of Rv0801 in *mycobacteria* revealed that it contains a vicinal oxygen chelate (VOC) domain, a glyoxalase-like domain, and binding sites for zinc and manganese ions. **(D)** Schematic diagram of *Rv0801* and *MSMEG_5827* gene clusters in *Mycobacterium* species. **(E)** Wild-type *M. smegmatis* (WT_MS) cells were treated with 2 mM MG for 3 h or left untreated. *MSMEG_5827* mRNA levels, normalized to *rpoB* mRNA, were determined by RT-qPCR to assess the induction following MG treatment. **(F)** Growth curves of wild-type *M. smegmatis* carrying an empty vector (MS_pAL) and *Rv0801* recombinant *M. smegmatis* (MS_*Rv0801*) in medium with or without 0.25 mM MG, measured by OD_600_ over 48 h. **(G)** Growth curves of WT_MS, *MSMEG_5827* knockout *M. smegmatis* Δ*MSMEG_5827* and complemented strain Δ*MSMEG_5827*(pHY-*MSMEG_5827*) in medium with or without 0.25 mM MG, measured by OD_600_ over 48 h. **(H)** Growth of MS_pAL and MS_*Rv0801* without treatment (Control) or treated with 2 mM MG for 24 h was analyzed using CFU counting assays on 7H9 agar plates. **(I)** Growth of WT_MS, Δ*MSMEG_5827*(pHY-*MSMEG_5827*) and Δ*MSMEG_5827* strains without treatment (Control) or treated with 0.5 mM MG for 24 h was analyzed using CFU counting assays on 7H9 agar plates. For all experiments, data from three independent experiments were shown. Only one representative image per experiment was presented. Two-tailed unpaired Student’s *t*-test was used for statistical analysis; **P < 0.01.

Bacterial glyoxalase system primarily consists of GloA and GloB. MG spontaneously reacts with GSH to form a hemithioacetal, which is converted by GloA into S-D-lactoylglutathione. This intermediate can regulate bacterial potassium ion efflux pumps, inducing K^+^ efflux and concurrent H^+^/Na^+^ influx, leading to cytoplasmic acidification (35). Intracellular acidification is proposed as a key response mechanism against MG toxicity, potentially triggering activation of DNA damage repair pathways. S-D-lactoylglutathione is further hydrolyzed to D-lactate by GloB, regenerating reduced GSH. Lactate is then oxidized to pyruvate via lactate dehydrogenase (**Figure 1A**).

To investigate the function of *Rv0801*, we first predicted its three-dimensional structure using SWISS-MODEL with a *Bacillus cereus* glyoxalase template (SMTL ID: 1ss4.1.A) (**Figure 1B**). The model revealed a conserved oxygen-binding domain and a glyoxalase-like domain, with sequence alignment confirming high conservation across *Mycobacterium* species (**Figure 1C**). To further explore its function, we heterologously expressed Rv0801 in *M. smegmatis* (**Supplementary Figure S1A**), whose ortholog is MSMEG_5827 (**Figure 1D**). Functional comparisons of both genes were then performed to assess their roles in MG detoxification.

To determine whether MSMEG_5827 response to MG stress, we assessed transcriptional dynamics of wild-type *M. smegmatis* exposed to 2 mM MG for 3 h. Results revealed a significant upregulation of *MSMEG_5827* (**Figure 1E**). To dissect the function of Rv0801, we expressed it in *M. smegmatis*, and monitored growth over 48 h in 7H9 medium containing 0.25 mM MG. While the empty vector control strains exhibited clear growth inhibition during the logarithmic phase, the Rv0801 recombinant strains grew normally (**Figure 1F**). Conversely, the *MSMEG_5827* knockout strains (**Supplementary Figure S1B**) displayed pronounced growth delay upon MG exposure compared to wild-type, with no defect in standard medium (**Figure 1G**). We further quantified bacterial survival after MG challenge using CFU counting assays. Strains were exposed to 0.5 or 2 mM MG in 7H9 liquid culture for 24 h, then plated for viability counts. The Rv0801 recombinant strains showed enhanced MG tolerance, while the *MSMEG_5827* knockout strains exhibited increased sensitivity (**Figures 1H, I**). Together, these findings establish Rv0801 and its homolog MSMEG_5827 as key components of the mycobacterial MG-detoxification machinery, with direct implications for bacterial fitness under carbonyl stress.

### 3.2 Rv0801-mediated MG detoxification process is mycothiol (MSH)-dependent

The canonical glyoxalase pathway relies on GSH as a cofactor for detoxifying MG. However, in GSH-deficient Gram-positive Firmicutes such as *Bacillus subtilis*, MG detoxification depends on the unique bacillithiol (BSH) (36). Similarly, *Mycobacteria* utilize mycothiol (MSH) as their primary redox buffer against oxidative stress (37), implying a potential role for MSH in MG detoxification.

**Figure 2:**
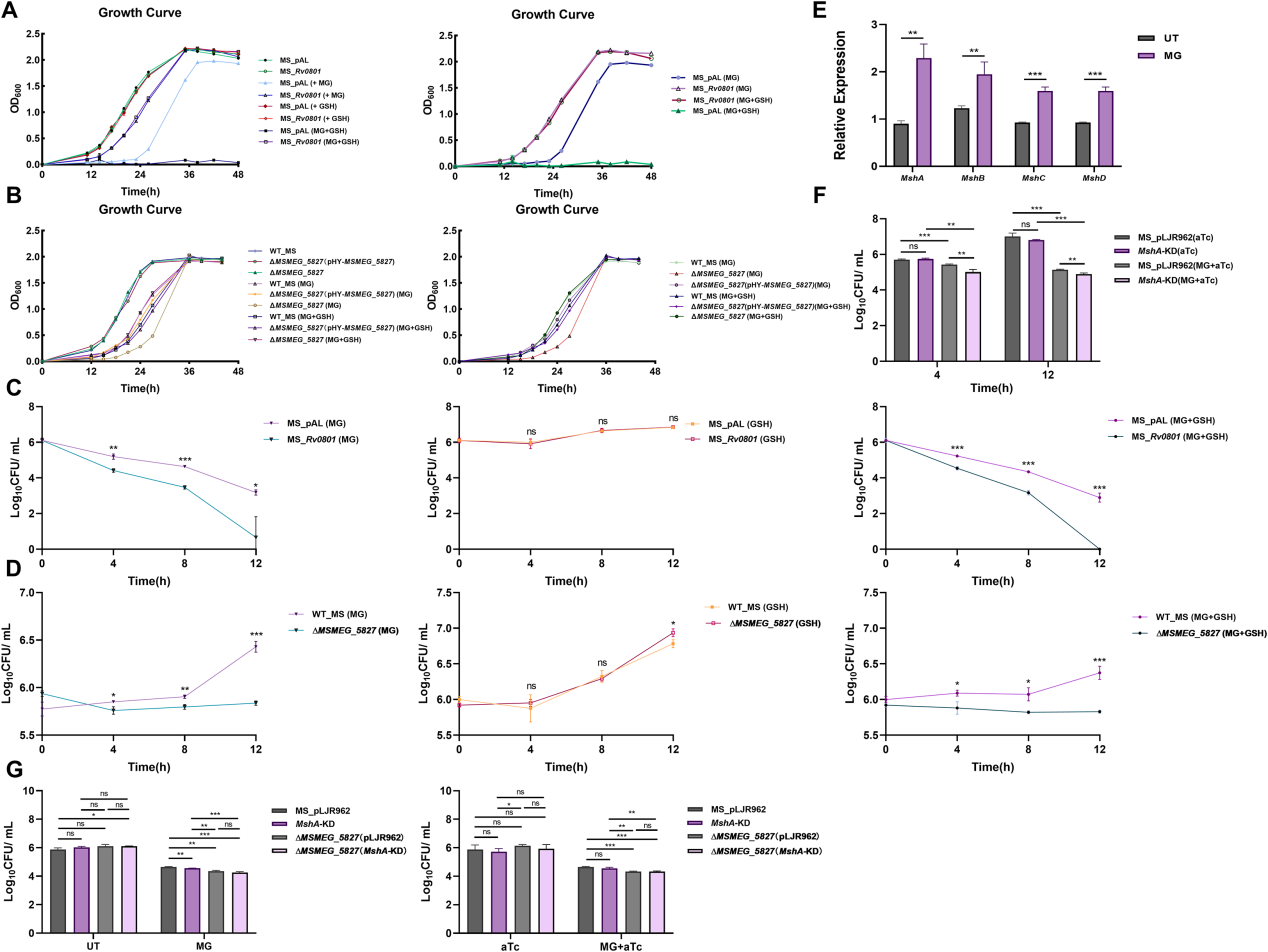
The detoxification of MG mediated by Rv0801 relies on MSH. **(A)** Growth curves of MS_pAL and MS_*Rv0801* in medium with or without 0.25 mM MG, measured by OD_600_ over 48 h. GSH (0.25 mM) was added at 12 h of bacterial growth. **(B)** Growth curves of WT_MS, Δ*MSMEG_5827* and *MSMEG_5827*(pHY-*MSMEG_5827*) *s*trains in medium with or without 0.25 mM MG, measured by OD_600_ over 48 h. GSH (0.25 mM) was added at 12 h of bacterial growth. **(C)** Growth of MS_pAL and MS_*Rv0801* without treatment (Control) or treated with 2 mM MG for 4, 8 or 12 h was analyzed using CFU counting assays on 7H9 agar plates with or without 2 mM GSH. **(D)** Growth of WT_MS and Δ*MSMEG_5827* without treatment (Control) or treated with 1 mM MG for 4, 8 or 12 h was analyzed using CFU counting assays on 7H9 agar plates with or without 1 mM GSH. **(E)** WT_MS cells were treated with 2 mM MG for 3 h or left untreated. *MshA*, *MshB*, *MshC* and *MshD* mRNA levels, normalized to *rpoB* mRNA, were determined by RT-qPCR to assess the induction following MG treatment. **(F)** Growth of empty vector strains (MS_pLJR962) and *MshA* knockdown mutants (*MshA*-KD) without treatment (Control) or treated with 1 mM MG for 4 or 12 hours was analyzed using CFU counting assays on 7H9 agar plates. **(G)** Growth of MS_pLJR962, *MshA*-KD, Δ*MSMEG_5827*(pLJR962) and Δ*MSMEG_5827*(*MshA*-KD) mutants without treatment (Control) or treated with 2 mM MG for 4 h was analyzed using CFU counting assays on 7H9 agar plates. For all experiments, data from three independent experiments were shown. Two-tailed unpaired Student’s *t*-test was used for statistical analysis; *P < 0.05, **P < 0.01, ***P < 0.001.

To assess the role of GSH in mycobacterial MG detoxification, we monitored bacterial growth under MG stress with or without GSH supplementation. The Rv0801 recombinant strains showed no alteration in growth kinetics upon addition of 0.25 mM GSH at 12 h during a 0.25 mM MG challenge (**Figure 2A**). In contrast, the empty vector control strains exhibited growth arrest in GSH-supplemented medium, while cultures without added GSH demonstrated initial growth inhibition followed by gradual recovery (**Figure 2A**). However, this does not exclude the effect of the plasmid itself on bacterial growth. For the *MSMEG_5827* knockout strains, MG exposure caused early growth retardation but permitted eventual recovery after 36 h (**Figure 2B**), suggesting that this gene product contributes quantitatively but not absolutely to detoxification capacity. Notably, both wild-type and complemented strains exposed to combined MG and GSH showed transient early growth inhibition, but recovered to levels comparable to those treated with MG alone by 36 h (**Figure 2B**). This implies functional independence from GSH for Rv0801- and MSMEG_5827-mediated MG detoxification pathways.

To further test this model, we performed CFU counting assays in liquid cultures treated with 1 mM or 2 mM MG, measuring viability after 4, 8 and 12 h. When exposed to GSH-supplemented solid media, no mitigation of MG-induced CFU counts disparities was observed between empty vector controls and Rv0801 recombinant strains (**Figure 2C**). A similar trend was observed in the *MSMEG_5827* knockout strains (**Figure 2D**). Collectively, these findings support that mycobacterial glyoxalase system operates through non-GSH-dependent mechanisms.

Previous studies revealed that exogenous GSH disrupts MSH-dominated redox homeostasis in *mycobacteria*, thereby inducing cytotoxicity (38). We therefore hypothesized that GSH may impose reductive stress that synergizes with MG-induced oxidative damage to inhibit bacterial proliferation. To investigate whether MSH is required for *mycobacteria* MG detoxification, we first examined the transcriptional levels of MSH biosynthetic genes (*MshA*, *MshB*, *MshC*, and *MshD*) in wild-type *M. smegmatis* after exposure to 2 mM MG for 3 h. All four genes were significantly upregulated following MG induction (**Figure 2E**), suggesting MG-driven transcriptional activation of the MSH biosynthetic pathway. To further dissect the functional role of MSH in this process, we generated an *MshA* knockdown strain (validated by RT-qPCR, **Supplementary Figure S2A**). When challenged with 1 mM MG, the knockdown strains showed markedly reduced survival compared to the wild-type in CFU assays at both 4 h and 12 h (**Figure 2F**), establishing MSH’s protective role against MG toxicity.

To clarify whether the MG detoxification pathways mediated by Rv0801 and its homolog MSMEG_5827 depend on MSH, we constructed a double mutant Δ*MSMEG_5827*(*MshA*-KD) (**Supplementary Figure S2B**), and compared its survival with the corresponding single mutants under 2 mM MG exposure for 4 h. Both the single mutant Δ*MSMEG_5827* and the double mutant Δ*MSMEG_5827*(*MshA*-KD) exhibited significantly lower CFUs than *MshA*-KD strain (**Figure 2G**). Notably, the double mutant did not exhibit increased sensitivity compared to the Δ*MSMEG_5827* single mutant (**Figure 2G**), suggesting that disruption of MSH biosynthesis does not further sensitize cells already deficient in *MSMEG_5827*. Collectively, these findings uncover a non-canonical glyoxalase system in *mycobacteria* where MSH acts as a critical cofactor synergizing with Rv0801 or MSMEG_5827 to detoxify MG, underscoring *mycobacteria*’s evolutionary adaptation to employ unique thiol-dependent redox defense mechanisms.

### 3.3 Rv0801 alleviates MG-induced carbonyl and oxidative stress

**Figure 3:**
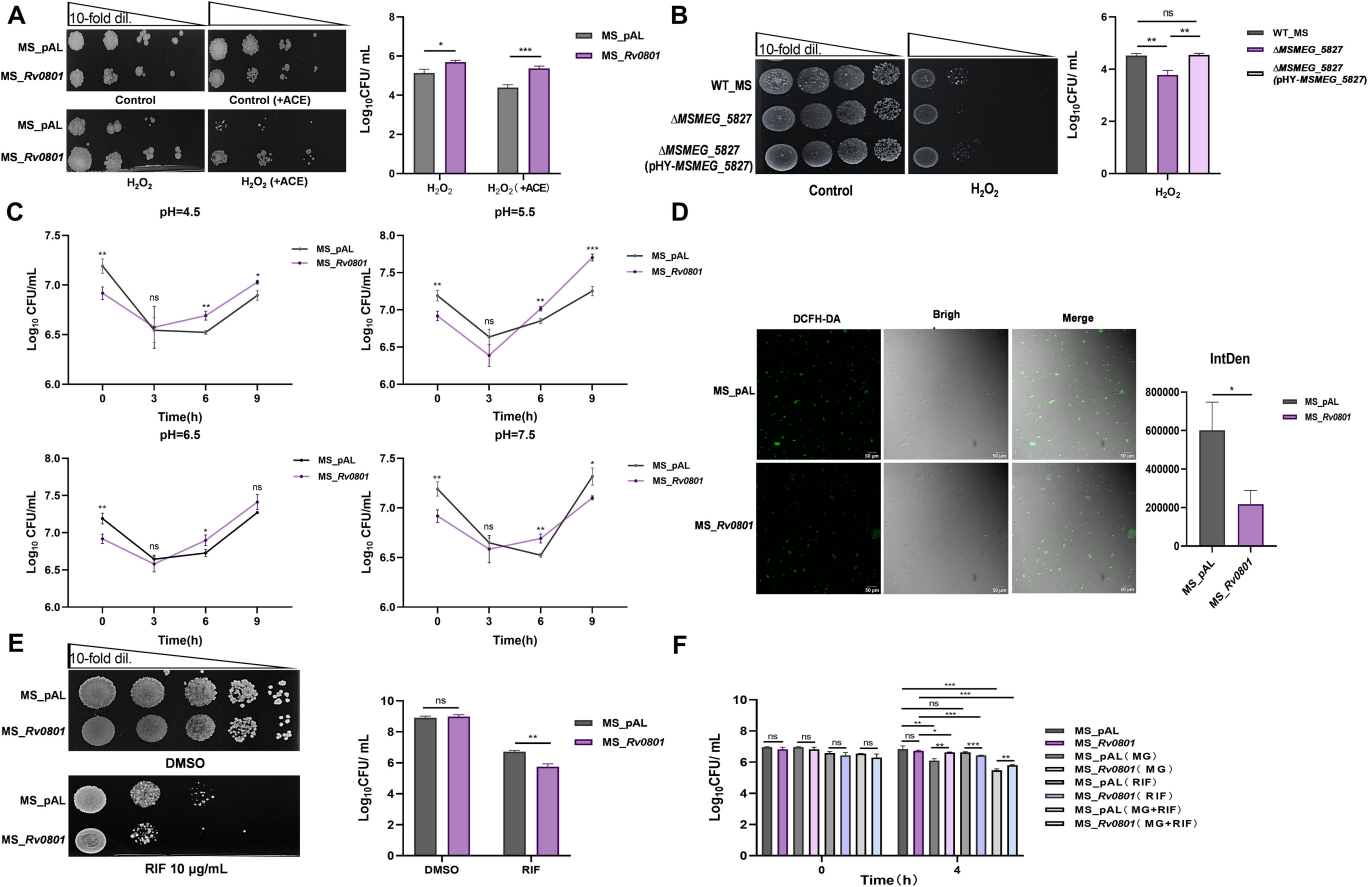
Rv0801 enhances tolerance to MG-induced carbonyl and oxidative stress. **(A)** Representative images of spot assays showing growth of MS_pAL and MS_*Rv0801* on 7H9 agar plates containing 0.07 mM H_2_O_2_. **(B)** Representative images of spot assays showing growth of WT_MS, Δ*MSMEG_5827* and Δ*MSMEG_5827*(pHY-*MSMEG_5827*) on 7H9 agar plates containing 0.07 mM H_2_O_2_. **(C)** Images show the viability of *M. smegmatis* at different pH conditions. The CFUs of MS_pAL and MS_*Rv0801* were determined at 0, 3, 6 and 9 hours under pH=4.5, pH=5.5, pH=6.5 and pH=7.5 conditions, respectively. **(D)** Detection of ROS levels in MS_pAL and MS_*Rv0801* following treatment with 2 mM MG for 3 h. At least 3 images for each strain were selected for ROS levels quantification. ImageJ was used to quantify fluorescence intensity. Images were acquired at 20× magnification; scale bar, 50 *μ*m. **(E)** Representative images of spot assays showing growth of MS_pAL and MS_*Rv0801* on 7H9 agar plates containing 10 *μ*g/mL rifampicin. **(F)** Growth of MS_pAL and MS_*Rv0801* with or without 1 mM MG induction for 4 h. Spot assays carried out on 7H9 agar plates containing rifampicin (5 *μ*g/mL) or not. Cell viability was determined by CFU counting assays. For all experiments, data from three independent experiments were shown. Only one representative image per experiment was presented. Two-tailed unpaired Student’s *t*-test was used for statistical analysis; *P < 0.05, **P < 0.01, ***P < 0.001.

MG accumulation typically elevates intracellular reactive oxygen species (ROS) levels, causing oxidative damage (39). To determine whether Rv0801 confers resistance to oxidative stress beyond MG detoxification, we compared the survival of empty vector controls and Rv0801 recombinant strains under 0.07 mM hydrogen peroxide (H_2_O_2_) exposure by performing CFU counting assays. Rv0801 recombinant strains exhibited a significant but moderate survival advantage under H_2_O_2_-induced oxidative stress (**Figure 3A**), while the *MSMEG_5827* knockout strains displayed increased H_2_O_2_ sensitivity (**Figure 3B**). These results establish functional conservation between Rv0801 and its homolog MSMEG_5827 in mediating mycobacterial resistance to diverse oxidative insults.

Acidic conditions trigger bacterial starvation responses that restrict carbon utilization and counteract reduction-related stress, which often overlaps with oxidative stress (40). To assess the role of Rv0801 in reductive stress resistance, we analyzed *M. smegmatis* survival across varying pH levels at 3 h, 6 h and 9 h by CFU counting assays. Notably, Rv0801 recombinant strains exhibited significantly higher CFUs than controls after 6 h of acidic exposure (**Figure 3C**). This suggests Rv0801 enhances bacterial resilience under adverse environmental conditions. To mechanistically link Rv0801 activity to ROS regulation under oxidative stress, intracellular ROS levels were quantified 3 h after 2mM MG treatment. Rv0801 recombinant strains demonstrated markedly lower ROS levels compared to controls (**Figure 3D**), indicating that Rv0801 activity mitigates oxidative damage by limiting ROS accumulation.

Given MG’s genotoxicity via DNA mutations (41–43) and ROS-driven DNA damage (44), which can drive rpoB mutations and rifampicin resistance (45), we next investigated Rv0801’s impact on antibiotic tolerance. Strikingly, Rv0801 recombinant strains displayed significantly higher susceptibility to rifampicin (10 *μ*g/mL) than controls (**Figure 3E**). When challenged concurrently with MG (1 mM) and rifampicin (5 *μ*g/mL), rifampicin-mediated killing was potentiated in both strains (**Figure 3F**). Notably, the survival benefit conferred by Rv0801 was compromised under these conditions (**Figure 3F**), suggesting that MG stress exacerbates rifampicin toxicity in this strain. These results indicate that Rv0801 is critical for genomic stability under metabolic stress through coordinated redox regulation.

### 3.4 Enhanced MG detoxification of Rv0801 recombinant *M. smegmatis* promotes bacterial fitness in THP-1 macrophages

**Figure 4:**
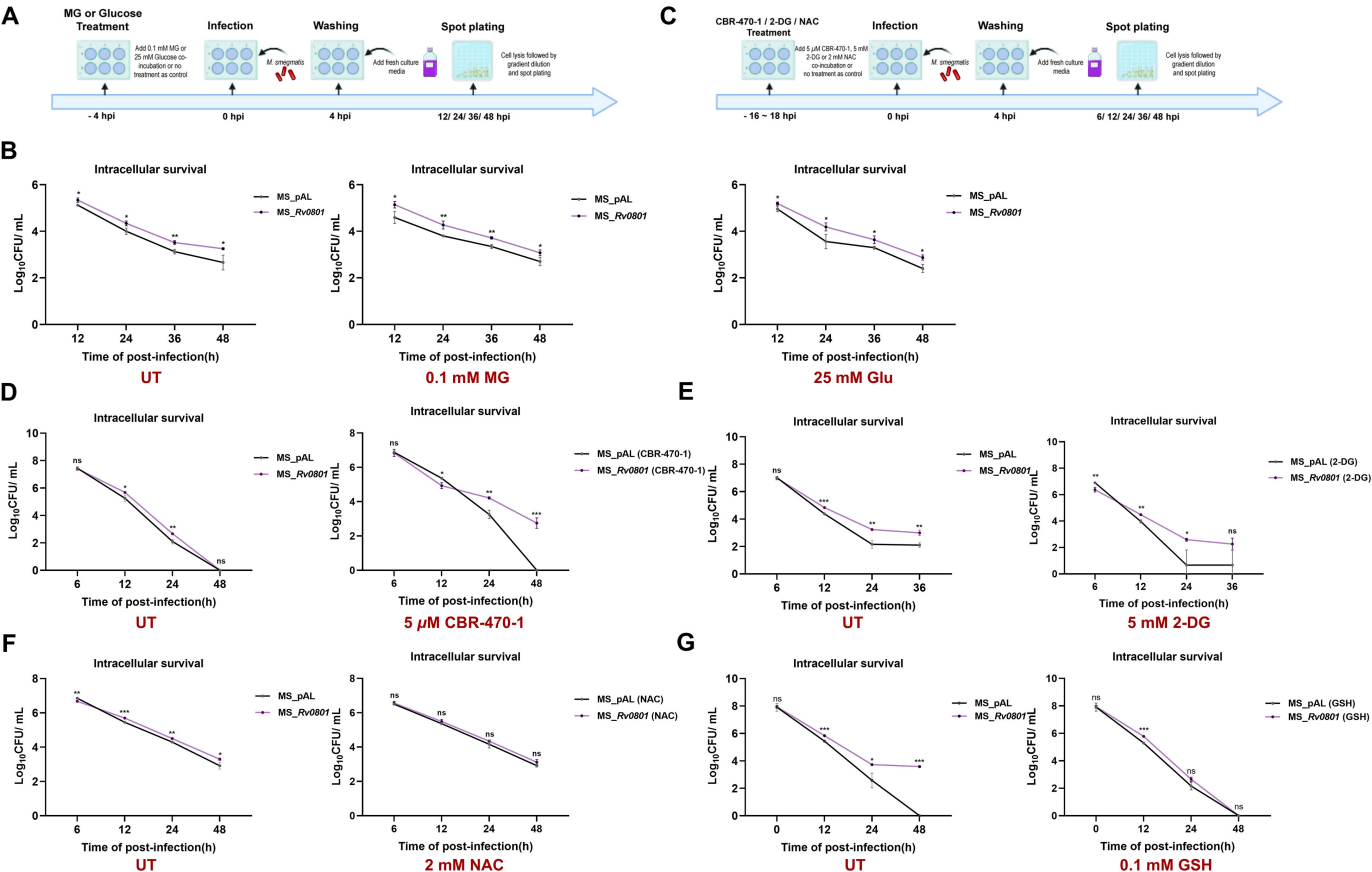
Rv0801 increases bacterial fitness in host by detoxifying MG. **(A)** Schematic workflow of bacterial infection of THP-1 macrophages under MG or glucose pretreatment conditions. **(B)** Intracellular survival of MS_pAL and MS_*Rv0801* in THP-1 macrophages pre-treated with 0.1 mM MG, 25 mM glucose for 4 h, or left untreated. Intracellular CFUs were enumerated at 12h, 24h, 36h and 48h post-infection (n = 3). **(C)** Schematic workflow of bacterial infection of THP-1 macrophages under CBR-470-1, 2-DG or NAC pretreatment condition. **(D)** Intracellular survival of MS_pAL and MS_*Rv0801* in THP-1 macrophages pre-treated with 5 *μ*M CBR-470-1 for 18 h. Intracellular CFUs were enumerated at 6 h, 12 h, 24 h and 48 h post-infection (n = 3). **(E)** Intracellular survival of MS_pAL and MS_*Rv0801* in THP-1 macrophages pre-treated with 5 mM 2-DG for 18 h. Intracellular CFUs were enumerated at 6 h, 12 h, 24 h and 36 h post-infection (n = 3). **(F)** Intracellular survival of MS_pAL and MS_*Rv0801* in THP-1 macrophages pre-treated with 2 mM NAC for 18 h. Intracellular CFUs were enumerated at 6 h, 12 h, 24 h and 48 h post-infection (n = 3). **(G)** Intracellular survival of MS_pAL and MS_*Rv0801* in THP-1 macrophages without treatment or treated with 0.1 mM GSH for 4 h after infection. Intracellular CFUs were enumerated at 12 h, 24 h and 48 h post-infection (n = 3). For all experiments, data from three independent experiments were shown. *P* values were calculated using two-tailed unpaired Student’s *t*-test; *P < 0.05, **P < 0.01, ***P < 0.001.

The metabolic shift to aerobic glycolysis in Mtb−infected macrophages elevates MG (8, 46), presenting a key challenge for intracellular survival. We therefore investigated whether the putative glyoxalase Rv0801 is required for Mtb to withstand this endogenous stress by performing macrophage infection assays. We found Rv0801 significantly enhanced bacterial survival in infected cells (**Figure 4B**). To dissect the link between MG detoxification and intracellular fitness, macrophages were pre-treated with 0.1 mM MG for 4 h to mimic a carbonyl−stressed host environment (**Figure 4A**). Under exogenous MG exposure, the survival advantage of the Rv0801 recombinant strains was further enhanced (**Figure 4B**). However, pre−treatment with 25 mM glucose—which elevates endogenous MG via glycolysis, did not widen the strain disparity (**Figures 4A, B**).

We next tested whether Rv0801 helps bacteria cope with host−derived MG produced during infection. Pre-treatment of THP-1 cells with 5 *μ*M CBR-470–1 for 18 h (**Figure 4C**), a protein kinase G (PKG) inhibitor that promotes glycolytic flux and endogenous MG accumulation (13), resulted in a sustained survival advantage for the Rv0801 recombinant strains at 48 h (**Figure 4D**). Conversely, inhibiting glycolysis with 2-deoxy-D-glucose (2-DG, 5 mM) markedly reduced the Rv0801-conferred survival benefit compared to the untreated group (**Figures 4C, E**).

The homeostasis of MG is tightly regulated by host cellular antioxidant defenses. To further determine whether Rv0801−mediated survival involves perturbing this balance, we pre-treated THP−1 macrophages with the ROS inhibitor N−acetylcysteine (NAC, 2 mM) for 18 h (**Figure 4C**) or exogenously supplemented with 0.1 mM GSH after 4 h infection. Both treatments abolished the intracellular survival advantage of the Rv0801 recombinant strains (**Figures 4F, G**), confirming its fitness benefit depends on altering host redox homeostasis.

### 3.5 Rv0801 impedes the KEAP1-NRF2 signaling pathway and suppresses inflammatory responses

The KEAP1-NRF2 pathway is a master regulator of cellular antioxidant defense. However, excessive NRF2 signaling suppresses expression of critical microbicidal effector molecules in bone marrow-derived macrophages (BMMs), impairing mycobacterial clearance (47).

**Figure 5:**
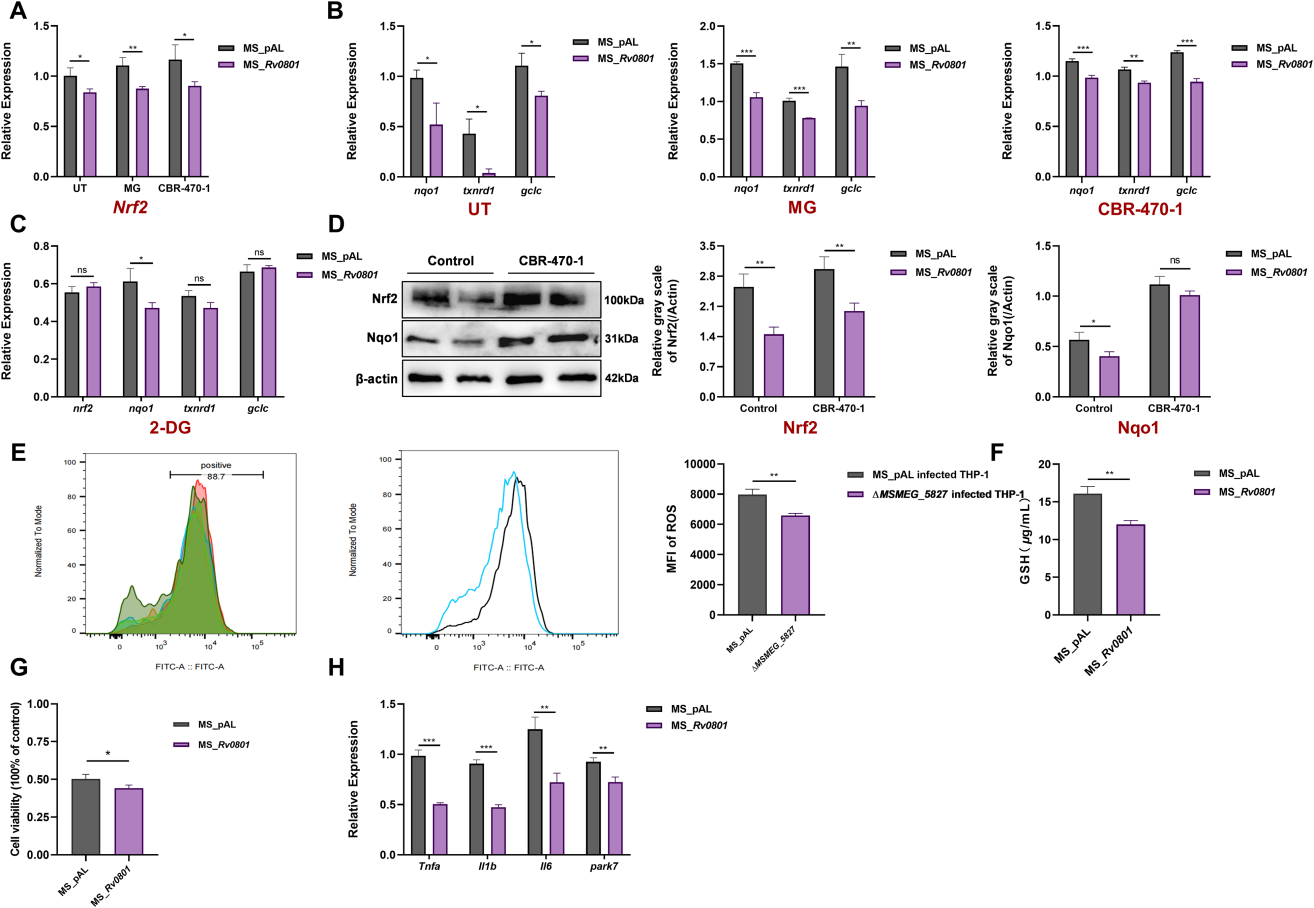
The MG-detoxifying function mediated by Rv0801 inhibits the KEAP1-NRF2 signaling pathway and attenuates immune inflammatory responses. **(A)** THP-1 cells were treated with 0.1 mM MG for 4 h or 5 *μ*M CBR-470-1 for 18 h before infection or left untreated. The increase of *nrf2* mRNA normalized to the *β-actin* mRNA levels in the same sample was determined by RT-qPCR. **(B)** THP-1 cells were treated with 0.1 mM MG for 4 h or 5 *μ*M CBR-470-1 for 18 h before infection or left untreated. The increase of *nqo1*, *txnrd1* and *gclc* mRNA 24 h post-infection was shown. **(C)** THP-1 cells were treated with 5 mM 2-DG for 18 h before infection or left untreated. The increase of *nrf2*, *nqo1*, *txnrd1* and *gclc* mRNA 24 h post-infection was shown. **(D)** Western blot analysis of Nrf2 and Nqo1 levels in THP-1 macrophages at 24 h post-infection. Quantitative analysis of the bands was carried out with ImageJ. **(E)** MS_pAL or Δ*MSMEG_5827* were used to infect 1×10^6^ THP-1 cells per milliliter at an MOI of 10:1. DCFH-DA was diluted with serum-free medium at a 1: 1000 ratio to a final concentration of 10 *μ*mol / L. At 24 h post-infection, cells were washed, and the diluted DCFH-DA solution was added. After incubation at 37°C for 30 minutes, flow cytometer was used for detection. The mean fluorescent intensity (MFI) of DCFH-DA-stained THP-1 cells reflected intracellular ROS levels. **(F)** The level of GSH in THP-1 cells infected by MS_pAL or MS_*Rv0801* was determined at 24 h post-infection. **(G)** Viability of THP-1 cells at 24 h post-infection was measured by CCK-8 assay. **(H)** The decrease of *tnf-a*, *il-1b*, *il-6* and *park7* mRNA 24 h after infection was shown. For all experiments, data from three independent experiments were shown. *P* values were calculated using two-tailed unpaired Student’s *t*-test; *P < 0.05, **P < 0.01, ***P < 0.001.

To determine whether Rv0801 influences the KEAP1-NRF2 axis, we first analyzed *Nrf2* transcriptional activity in THP-1 macrophages infected with Rv0801 recombinant strains. Compared to empty vector controls, Rv0801 significantly suppressed *Nrf2* mRNA expression (**Figure 5A**), accompanied by reduced transcription of antioxidant genes *nqo1*, *gclc*, and *txnrd1* (**Figure 5B**). Pre-treatment with 0.1 mM MG or 5 *μ*M CBR-470-1 further depressed their transcriptional output in Rv0801 recombinant strains-infected cells (**Figure 5B**), implying carbonyl stress synergizes with Rv0801 activity to dampen the host antioxidant response. Conversely, inhibition of glycolysis with 5 mM 2−DG abolished this suppression (**Figure 5C**). At the protein level, infection with the Rv0801 recombinant strains reduced NRF2 and its downstream target Nqo1 (**Figure 5D**). However, in cells pre-treated with CBR−470−1, the difference in Nqo1 protein levels between the Rv0801 recombinant and control strains was no longer observed (**Figure 5D**). This absence of a difference likely results from CBR−470−1 pretreatment fundamentally rewiring host cell metabolism, thereby obscuring the strain−specific effect. Next, we found out infection with *MSMEG_5827* deletion mutants reduced intracellular ROS levels versus vector controls (**Figure 5E**). This indicates a role for MSMEG_5827 in modulating the host redox environment. Consistent with a perturbed redox state, Rv0801 recombinant strains infection also significantly decreased intracellular GSH pools (**Figure 5F**).

To evaluate the effect of Rv0801 on host cell viability, we assessed cell survival rates after 24 h infection. Results show Rv0801 recombinant bacteria induced cytotoxicity in THP-1 macrophages (**Figure 5G**) and suppressed mRNA expression of pro-inflammatory cytokines *tnf-α*, *il-1b*, *il-6* and the oxidative stress sensor *park7*, at 24 h post-infection (**Figure 5H**). Collectively, these findings indicate that by detoxifying host−derived MG, Rv0801 helps maintain KEAP1−mediated repression of NRF2, thereby blunting the antioxidant response. This suppression, coupled with diminished GSH and pro−inflammatory signaling, creates an immunosuppressive niche that favors mycobacterial intracellular persistence.

## 4 Discussion

As a potent antimicrobial metabolite, MG presents a critical barrier for intracellular pathogens. Here, we uncover how Mtb adapts to this endogenous MG stress. We demonstrate that Rv0801, a putative MSH-dependent glyoxalase, is a critical metabolic effector that enhances bacterial fitness under carbonyl and oxidative stress. Rv0801 ensures fitness not merely by resisting individual stressors, but by acting as a metabolic guardian, detoxifying MG to maintain redox homeostasis. This homeostatic function underpins its pronounced survival advantage during sustained or multifactorial stress where MG-driven damage accumulates.

**Figure 6:**
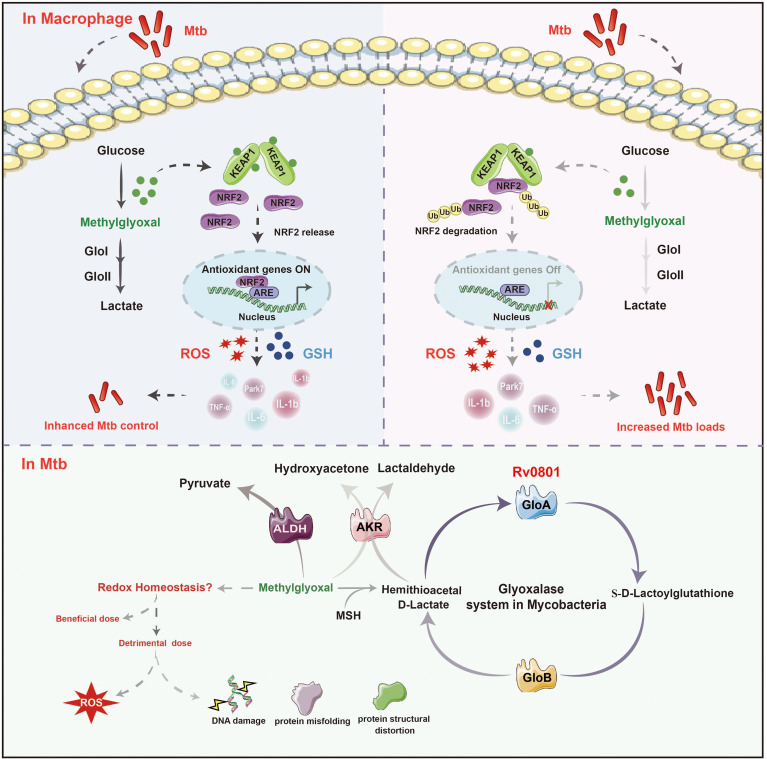
Schematic depicting the role of Rv0801 in host-pathogen interaction. The putative glyoxalase Rv0801 detoxifies MG in an MSH-dependent manner, enhancing bacterial tolerance to carbonyl stress. This function is critical for bacterial fitness inside macrophages. Concomitantly, MG detoxification by Rv0801 suppresses the activation of the host KEAP1-NRF2 pathway and inhibits pro-inflammatory cytokine production, leading to an immunosuppressive microenvironment that favors bacterial survival. The model also incorporates alternative MG detoxification routes in Mtb, including those mediated by aldehyde dehydrogenase (ALDH) and aldo-keto reductase (AKR). In the schematic, solid arrows denote direct metabolic conversions; dashed arrows represent associated biological processes or functional outcomes. Within the host-pathogen interaction layer (upper section), varying color intensities of the arrows indicate the relative strength or dominance of the depicted relationships.

Mechanistically, Rv0801 orchestrates a dual-pathway interference within host macrophages. By detoxifying MG, it suppresses the KEAP1-NRF2 antioxidant pathway and dampens pro-inflammatory cytokines production. This coordinated suppression subverts macrophage defenses. The central role of redox perturbation is confirmed by our functional assays: quenching host oxidative stress potential (via NAC) or restoring reducing equivalents (via GSH supplementation) abolished Rv0801’s survival advantage. Thereby, Rv0801 fosters an immunosuppressive niche that promotes bacterial persistence. Collectively, our findings, summarized in **Figure 6**, establish Rv0801 as a master metabolic effector that rewires the host immune landscape.

MG accumulation under metabolic stress fuels AGEs formation and inflammation. Epidemiological studies indicate a 2-3-fold higher tuberculosis risk in diabetic patients, who often exhibit elevated MG levels (48, 49). Notably, high−glucose pre−treatment did not widen the bacterial survival disparity, suggesting in glucose−rich environments, increased host antimicrobial activity may counterbalance MG toxicity. This highlights the complexity of metabolic−immune interactions governing infection outcomes. We thus propose that MG is a pivotal molecular signal dictating pathogen adaptability and host defense equilibrium. This balance is centrally regulated by the glyoxalase system and the KEAP1-NRF2 axis. Intriguingly, the outcome of modulating this axis is context-dependent. The probiotic *Bifidobacterium longum* Z1 enhances cellular detoxification and antioxidant defenses by activating the glyoxalase system and NRF2 signaling (50), whereas Mtb subverts it: through Rv0801-mediated MG detoxification, it attenuates KEAP1-NRF2 activity in macrophages, disabling a key host defense. This strategic hijacking of a conserved stress-response module underscores the sophistication of metabolic cross−talk in infection.

While this study establishes Rv0801’s dual roles in MG detoxification and immune modulation, unresolved questions remain. First, the substrate specificity of Rv0801 requires validation to determine whether it metabolizes other reactive carbonyl species (e.g., formaldehyde, glyoxal). Second, the mechanism of KEAP1-NRF2 suppression requires clarification. Specifically, it is unclear whether Rv0801 acts directly on the pathway components (e.g., via protein modification) or indirectly by redox-state modulation. Additionally, while Rv0801-mediated MG detoxification alleviates host cytotoxicity, it paradoxically suppresses immune responses to favor bacterial survival. This suggests that MG itself may function as a danger signal whose removal dysregulates host defense pathways. Furthermore, our *in vitro* macrophage model, while valuable, does not fully recapitulate the complex *in vivo* lung microenvironment. Future studies must address pulmonary infection dynamics in animal models of metabolic stress.

This work represents the first demonstration of an MSH-dependent glyoxalase system central to Mtb stress resistance, expanding our understanding of the metabolic-immune regulatory networks governing host-pathogen interactions. The discovery of this cross-kingdom regulatory strategy, mediated by a bacterial effector, suggests that targeting such pathways could yield innovative therapeutic strategies. Specifically, exploiting Mtb’s unique MSH-dependent mechanism could circumvent the drug resistance challenges associated with conventional antimycobacterial therapies.

## Data Availability

The original contributions presented in the study are included in the article/**Supplementary Material**. Further inquiries can be directed to the corresponding authors.
