# Supplementary material for: A putative Mycobacterium tuberculosis glyoxalase Rv0801 promotes bacterial fitness by alleviating methylglyoxal stress and blunts NRF2-mediated antioxidant defenses
Source: Front Immunol. 2026 Feb 27;17:1745502. doi: 10.3389/fimmu.2026.1745502 (PMC12982171; doi:10.3389/fimmu.2026.1745502)
Supplement: Supplementary file 1 [file Table1.docx]

***Supplementary Material***

1. **Supplementary Figures and Tables**

**1.1 Supplementary Figures**

**
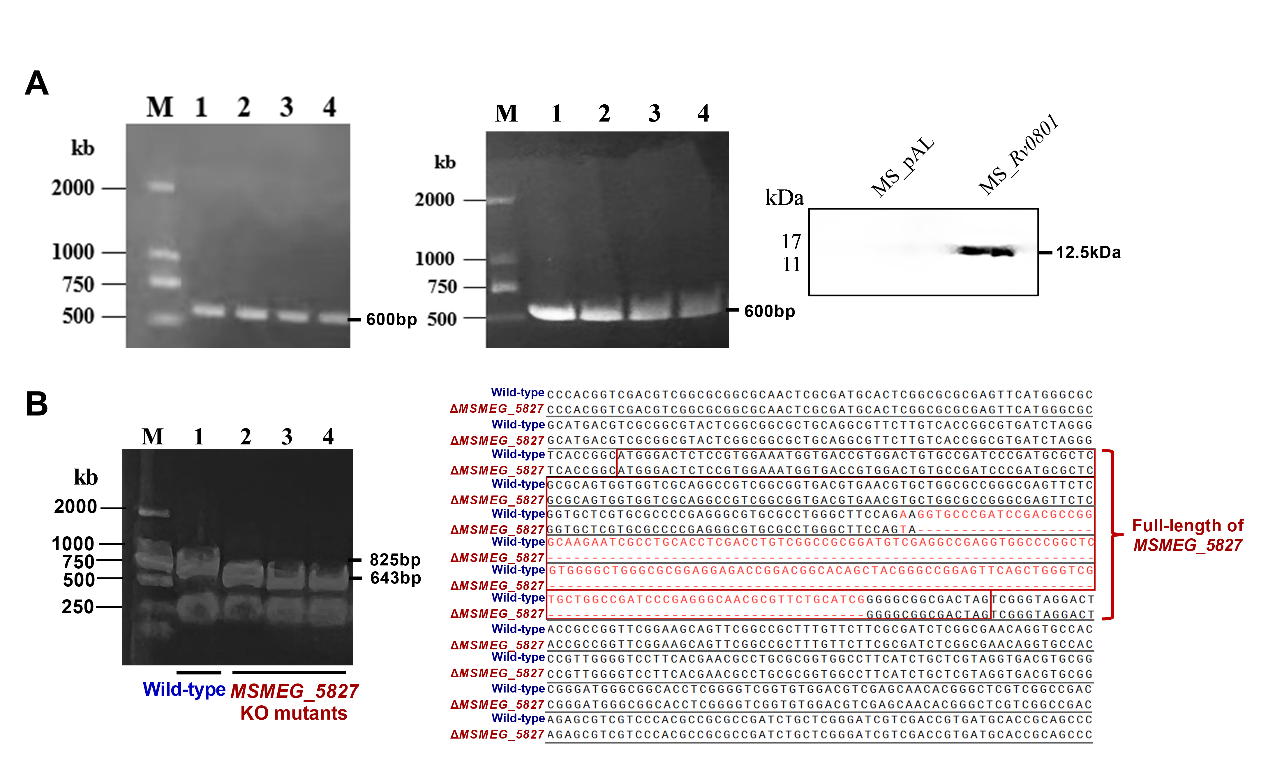
**

**Supplementary Figure 1. Construction of Rv0801 recombinant *M. smegmatis* and** ***MSMEG_5827* knockout mutant.**

(A) Rv0801 recombinant *M. smegmatis* construction. *Rv0801* was ligated with the pALACE plasmid and successfully expressed in *E.coli* DH5α which was verified by bacterial liquid PCR with lanes 1-4 of recombinant strains and lane M of DL 2000 marker (The first electropherogram). Then the recombinant plasmid was successfully transferred into *M. smegmatis* (The second electropherogram). Rv0801 protein expression was assessed by Western blotting with size of approximately 12.5 kDa in *Rv0801* recombinant *M. smegmatis.*

(B) *MSMEG_5827* knockout mutant construction. CRISPR-Cas12a with assisted recombineering successfully deletes *MSMEG_5827* with lane M of DL 2000 marker, lane 1 of wild-type *M. smegmatis* and lanes 2-4 of *MSMEG_5827* knockout mutants. Sequence alignment result showed successful knockout of *MSMEG_5827* gene. The upper line is original DNA sequence of *MSMEG_5827* including upstream and downstream of 300 base pairs and the bottom line is sequencing result.


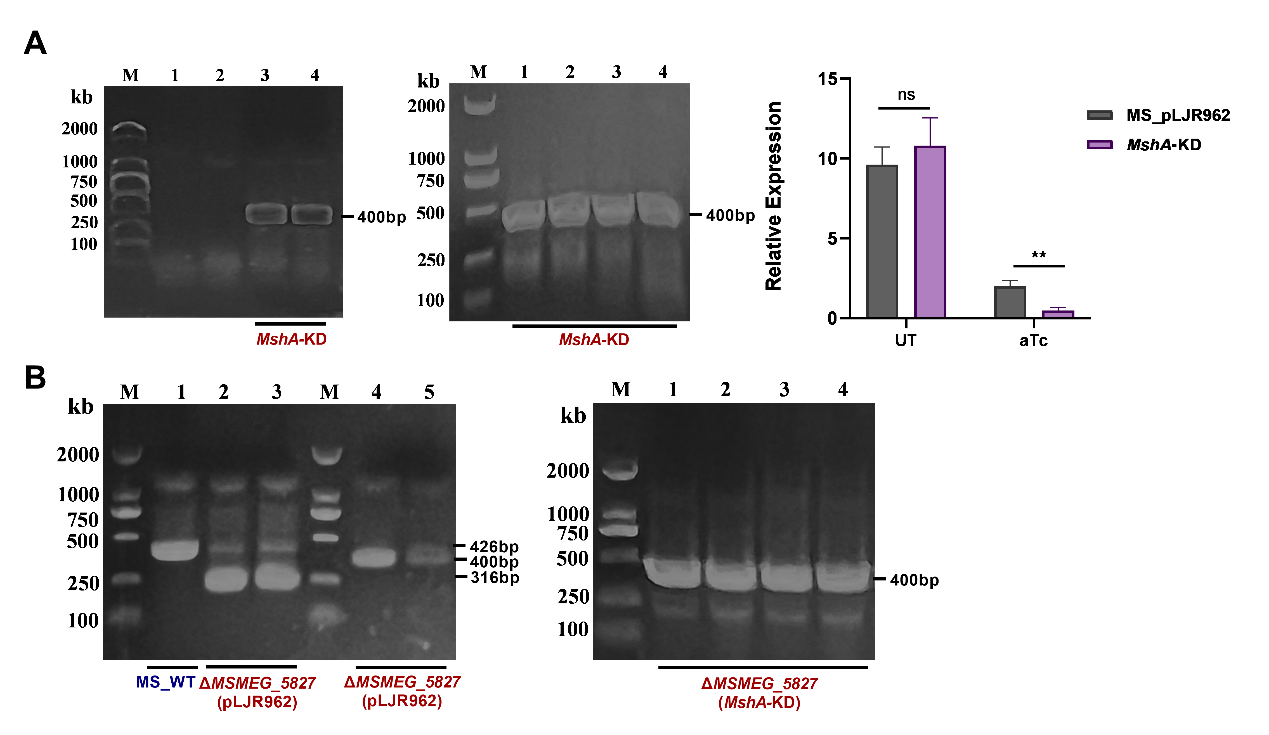


**Supplementary Figure 2. Construction of *MshA* knockdown and Δ*MSMEG_5827*(*MshA-*KD) mutants.**

(A) *MshA* knockdown mutants construction. Recombinant plasmid was verified by bacterial liquid PCR with lanes 3-4 of *MshA*-KD, lane M of DL 2000 marker (The first electropherogram). Then the recombinant plasmid was successfully transferred into *M. smegmatis* (The second electropherogram). mRNA expression level of *MshA* in wild-type *M. smegmatis* carrying an empty vector (MS_pLJR962) and *MshA* knockdown strains (*MshA*-KD) was verified by quantitative real-time PCR. Two-tailed unpaired Student’s *t*-test was used for stastistical analysis. ns P＞0.05, ** P < 0.01.

(B) Δ*MSMEG_5827*(*MshA-*KD) mutants construction. Δ*MSMEG_5827*(pLJR962) strains verified by bacterial liquid PCR with lane 1 of MS_WT, lanes 2-3 of Δ*MSMEG_5827*(pLJR962) strains using *MSMEG_5827* P1 and P4 primers, lanes 4-5 of Δ*MSMEG_5827*(pLJR962) strains using plasmid pLJR962 primers, lane M of DL 2000 marker (The first electropherogram). Construction of Δ*MSMEG_5827*(*MshA-*KD) mutants verified by bacterial liquid PCR with lane 1-4 of Δ*MSMEG_5827*(*MshA-*KD) mutants, lane M of DL 2000 marker (The second electropherogram).

**1.2 Supplementary Tables**

**Supplementary Table 1.** Strains, plasmids and primers used in this study. The purpose of each primer in the present study is also mentioned for easy reference.

| **Strains** | **Description** | |
| --- | --- | --- |
| *M. smegmatis* | Wild type *M. smegmatis* mc^2^155 | |
| MS_pAL | Wild type *M. smegmatis* mc^2^155 transformed with empty vector pALACE | |
| MS_*Rv0801* | Wild type *M. smegmatis* mc^2^155 transformed with vector pALACE-*Rv0801* | |
| Δ*MSMEG_5827* | Deletion in amino acids 71-107 of *MSMEG_5827* in *M. smegmatis* for *MSMEG_5827* knockout strain construction | |
| Δ*MSMEG_5827*(pHY-*MSMEG_5827*) | Δ*MSMEG_5827* transformed with vector pHY-*MSMEG_5827* for complemented strain construction by using pHY plasmid | |
| MS_pLJR962 | Wild type *M. smegmatis* mc^2^155 transformed with vector pLJR962 | |
| *MshA*-KD | Wild type *M. smegmatis* mc^2^155 transformed with *MshA* CRISPRi plasmid for *MshA* knockdown strain construction by using pLJR962 plasmid | |
| Δ*MSMEG_5827*(pLJR962) | Δ*MSMEG_5827* transformed with vector pLJR962 | |
| Δ*MSMEG_5827*(*MshA*-KD) | Δ*MSMEG_5827* transformed with *MshA* CRISPRi plasmid for *MSMEG_5827* knockout *MshA* knockdown strain construction | |
| *E.coli* DH5α | The strain used in vector proliferation | |
| **Plasmids** | **Description** | |
| pALACE | An inducible plasmid with His-tag used for recombinant strain construction | |
| pJV53-Cpf1 | A plasmid used for knockout in *M. smegmatis* | |
| pCR-Hyg | A plasmid used for knockout in *M. smegmatis* | |
| pHY | An integrative plasmid used for gene complement in *M. smegmatis* and conferring kanamycin (kan) resistance | |
| pLJR962 | A plasmid used for knockdown in *M. smegmatis* | |
| **Primer name** | **Sequence (5' to 3')** | **Purpose** |
| pAL_*Rv0801*_F | ATCGAGGGCCGCGGATCCGTTTCTTTCCGCCGAGCTATC | Construction of Rv0801 recombinant *M. smegmatis* |
| pAL_*Rv0801*_R | CGAGGTCGACGGTATCGATCGCCTCGTTATTGACCCG |  |
| pALACE_F | AGCGTCGACCATTCGGGAT | PCR confirmation of Rv0801 recombinant *M. smegmatis* |
| pALACE_R | GCTTGTCTCCGAATCCAACTGG |  |
| *MSMEG_5827*_P1 | TCGACGATCGGGCCGATG | Construction of  *MSMEG_5827* knockout strain |
| *MSMEG_5827*_P2 | CCGACTAGTCGCCGCCCCTACTGGAAGCCCAGGCGCAC |  |
| *MSMEG_5827*_P3 | GTGCGCCTGGGCTTCCAGTAGGGGCGGCGACTAGTCGG |  |
| *MSMEG_5827*_P4 | AGAGCCTGCCCGAGGTGT |  |
| crRNA_5827_F | ATTCGGTGCTCGTGCGCCCCGAGGA |  |
| crRNA_5827_R | AGCTTCCTCGGGGCGCACGAGCACCGAATCT |  |
| pHY-*MSMEG_5827*_F | CGCCACGTGGGGCCCAAGCTTC CAACTACCCGGAGCGCCACG | Construction of  *MSMEG_5827* complemented strain |
| pHY-*MSMEG_5827*_R | ACCGGCGCTCAGCTGGAATTCCGAAGAACAAAGCGGCCGAAC |  |
| pLJR962_F | CCGAAATCAACACCCTGTC | PCR confirmation of knockdown strains |
| pLJR962_R | GGGAAACGCCTGGTATCTTT |  |
| sgRNA_*MshA*_F | GGGAGAACGCCACGATCTTCTGGTCGG | Construction of *MshA* knockdown strain |
| sgRNA_*MshA*_R | AAACCCGACCAGAAGATCGTGGCGTTC |  |
| *rpoB*_RT_F | TCGATGTCACTGTCCTTCTCGGATC | Real Time PCR for  *rpoB* expression |
| *rpoB*_RT_R | GACCGTCTGGCTCTTGATCTC |  |
| *MSMEG_5827*_RT_ F | GGAAATGGTGACCGTGGACT | Real Time PCR for  *MSMEG_5827* expression |
| *MSMEG_5827*_RT_ R | GAGGTGCAGGCGATTCTTG |  |
| *MshA*_RT_F | CTTCCCACCGACCAGAAGAT | Real Time PCR for  *MshA* expression |
| *MshA*_RT_R | GTCACCCGGTCACTGATACC |  |
| *MshB*_RT_F | GAGTGAACGCCGAGGACAT | Real Time PCR for  *MshB* expression |
| *MshB*_RT_R | AAGCGCGATGTTGTTGGAC |  |
| *MshC*_RT_F | GTACCCCGACGTGTACTTCC | Real Time PCR for  *MshC* expression |
| *MshC*_RT_R | GTCGAGTTGATCGGACTTGC |  |
| *MshD*_RT_F | GAGATCCTGCGGGTCAACA | Real Time PCR for  *MshD* expression |
| *MshD*_RT_R | TCGTGGATCTTGGTCCAGTG |  |
| β-actin_F | CATGTACGTTGCTATCCAGGC | Real Time PCR for  β-actin expression |
| β-actin_R | CTCCTTAATGTCACGCACGAT |  |
| *nrf2*_F | TCAGCGACGGAAAGAGTATGA | Real Time PCR for  *nrf2* expression |
| *nrf2*_R | CCACTGGTTTCTGACTGGATGT |  |
| *nqo1*_F | GAAGAGCACTGATCGTACTGGC | Real Time PCR for  *nqo1* expression |
| *nqo1*_R | GGATACTGAAAGTTCGCAGGG |  |
| *gclc*_F | GGAGACCAGAGTATGGGAGTT | Real Time PCR for  *gclc* expression |
| *gclc*_R | CCGGCGTTTTCGCATGTTG |  |
| *txnrd1*_F | ATATGGCAAGAAGGTGATGGTCC | Real Time PCR for  *txnrd1* expression |
| *txnrd1*_R | GGGCTTGTCCTAACAAAGCTG |  |
| *il-1b*_F | ATGATGGCTTATTACAGTGGCAA | Real Time PCR for  *il-1b* expression |
| *il-1b*_R | GTCGGAGATTCGTAGCTGGA |  |
| *il-*6_F | ACTCACCTCTTCAGAACGAATTG | Real Time PCR for  *il-*6 expression |
| *il-*6_R | CCATCTTTGGAAGGTTCAGGTTG |  |
| *tnf-a*_F | CCTCTCTCTAATCAGCCCTCTG | Real Time PCR for  *Tnf-a* expression |
| *tnf-a*_R | GAGGACCTGGGAGTAGATGAG |  |
| *park7*_F | GTAGCCGTGATGTGGTCATTT | Real Time PCR for  *park7* expression |
| *park7*_R | CTGTGCGCCCAGATTACCT |  |
